# Variable expression levels of keratin and vimentin reveal differential EMT status of circulating tumor cells and correlation with clinical characteristics and outcome of patients with metastatic breast cancer

**DOI:** 10.1186/s12885-015-1386-7

**Published:** 2015-05-13

**Authors:** Hara Polioudaki, Sofia Agelaki, Rena Chiotaki, Eleni Politaki, Dimitris Mavroudis, Alexios Matikas, Vassilis Georgoulias, Panayiotis A Theodoropoulos

**Affiliations:** 1 Department of Biochemistry, School of Medicine, University of Crete, Heraklion, Greece; 2 Laboratory of Τumor Cell Βiology, School of Medicine, University of Crete, Heraklion, Greece; 3 Department of Medical Oncology, University General Hospital of Heraklion, Heraklion, Greece

**Keywords:** Circulating tumor cells, EMT, Breast cancer, Keratin expression levels, Fluorescence levels of cell markers, Vimentin/keratin ratio

## Abstract

**Background:**

CTCs expressing variable levels of epithelial and mesenchymal markers in breast cancer have previously been reported. However, no information exists for keratin expression levels of CTCs in association with disease status, whereas assays for the characterization of transitional EMT phenotypes of CTCs in breast cancer are rather lacking. We investigated the correlation between keratin expression of CTCs and patients’ outcome and characterized the EMT status of CTCs via the establishment of a numerical “ratio” value of keratin and vimentin expression levels on a single cell basis.

**Methods:**

Keratin expression was evaluated in 1262 CTCs from 61 CTC-positive patients with metastatic breast cancer, using analysis of images obtained through the CellSearch System. For the determination of vimentin/keratin (vim/K) ratios, expression levels of keratin and vimentin were measured in cytospin preparations of luminal (MCF-7 and T47D) and basal (MDA.MB231 and Hs578T) breast cancer cell lines and 110 CTCs from 5 CTC-positive patients using triple immunofluorescence laser scanning microscopy and image analysis.

**Results:**

MCF-7 and T47D displayed lower vim/K ratios compared to MDA.MB231 and Hs578T cells, while MCF-7 cells that had experimentally undergone EMT were characterized by varying intermediate vim/K ratios. CTCs were consisted of an heterogeneous population presenting variable vim/K values with 46% of them being in the range of luminal breast cancer cell lines. Keratin expression levels of CTCs detected by the CellSearch System correlated with triple negative (p = 0.039) and ER-negative (p = 0.025) breast cancer, and overall survival (p = 0.038).

**Conclusions:**

Keratin expression levels of CTCs correlate with tumor characteristics and clinical outcome. Moreover, CTCs display significant heterogeneity in terms of the degree of EMT phenotype that probably reflects differential invasive potential. The assessment of the vim/K ratios as a surrogate marker for the EMT status of CTCs merits further investigation as a prognostic tool in breast cancer.

**Electronic supplementary material:**

The online version of this article (doi:10.1186/s12885-015-1386-7) contains supplementary material, which is available to authorized users.

## Background

CTCs are typically identified based on the expression of epithelial markers such as keratins, EpCAM (Epithelial Cell Adhesion Marker) and the absence of the common leukocyte marker CD45. Keratins are differentially expressed among different breast cancer cell lines and are down-regulated during metastatic spread and progression in breast cancer [1]. Moreover, it has been suggested that modulation of keratins due to Epithelial-to-Mesenchymal Transition (EMT) occurs frequently in CTCs of breast cancer patients and may be associated with an unfavorable outcome [1].

EMT is a process that generates invasive cells with the ability to enter the blood stream ([2] and references therein). It has been suggested that CTCs undergo EMT in order to migrate to distant organs [3-5]. During EMT, epithelial cells display decreased expression of epithelial markers (loss of epithelial keratins, including 8, 18 and 19, and downregulation of E-cadherin, occludins, claudins and desmoplakin) and acquire mesenchymal traits (up-regulation of vimentin, N-cadherin, fibronectin, alpha-smooth muscle actin). Vimentin filaments support the extension of tubulin-based microtentacles, which are promoted by EMT and enhance endothelial engagement [6,7]. Human cancer cells induced to undergo EMT have been shown to exhibit stem cell–like properties and increased metastatic potential [8].

Genome wide transcriptional analysis of human breast cancer cell lines has revealed a subgroup of cells with increased expression of EMT markers and high invasive potential, termed basal B/mesenchymal. These cells display a “mesenchymal” gene expression profile in contrast to a second subcategory, the luminal breast cancer cells, which exhibit poor invasive capability, low expression of EMT markers and bear an “epithelial” gene expression profile. Basal A breast cancer cells represent a third group with intermediate basal/luminal characteristics [9].

Using RT-PCR, Aktas et al. [3] reported that 62% of CTCs were positive for at least one EMT marker, whereas CTCs isolated by CELLection™Dynabeads coated with the monoclonal antibody toward EpCAM were negative for both keratins and CD45 [4], but positive for vimentin and fibronectin in 34% of patients with breast cancer. Although the expression of mesenchymal markers indicates that a cell may undergo EMT, it does not really determine the extent to which epithelial cells are engaged in the EMT process.

In a recent study, using a quantifiable, dual-colorimetric RNA–in situ hybridization assay for epithelial and mesenchymal transcripts, Yu et al. [5] defined five categories of CTCs ranging from exclusively epithelial (E) to intermediate (E > M, E = M, M > E) and exclusively mesenchymal (M). Forty-one percent of patients with metastatic breast cancer were scored positive for CTCs with EMT features; CTCs from patients with lobular type cancers (typically ER+/PR+) were predominantly epithelial, whereas those from the TN (Triple Negative) were predominantly mesenchymal.

In this study, we propose a new approach for the designation of EMT status of CTCs, based on the quantification of fluorescence intensity of keratin and vimentin on a single cell basis and the generation of a numerical ‘ratio’ value corresponding to their relative expression. “Epithelial” (MCF-7, T47D) and “mesenchymal” (Hs578T, MDA.MB231) breast cancer cell lines and “epithelial” (MCF-7) cells during experimentally induced EMT were employed as controls for the standardization of EMT ratio range. Furthermore, we present data that reveal a correlation between keratin expression levels of CTCs and patients’ clinical characteristics and disease outcome.

## Methods

### Cell lines and treatments

#### Culture conditions

MCF-7 (mammary adenocarcinoma), T47D (ductal breast epithelial tumor), MDA.MB231 and Hs578T (human breast carcinoma) cell lines were obtained from American Type Tissue Culture Collection (Manassas, VA). MCF-7 cells were cultured in Dulbecco’s modified Eagle’s medium (DMEM) plus 0.2 U/ml insulin, T47D in RPMI 1640 medium plus 0.2 U/ml insulin, MDA.MB231 and Hs578T in DMEM medium at 37°C in a humidified atmosphere containing 5% CO_2_.

Culture media were purchased from Biochrom (Berlin, Germany) and were supplemented with 10% heat-inactivated fetal bovine serum, penicillin and streptomycin.

#### EGF treatment

For the induction of Epithelial-to-Mesenchymal Transition, MCF-7 cells were treated with 100 ng/ml Epidermal Growth Factor (EGF) in low serum (0.1% FBS) DMEM with 1% penicillin/streptomycin, as described [10].

#### Cytospin preparation of cultured cells

Cells were harvested by trypsinization, washed with PBS and aliquots of 500000 cells were centrifuged at 2000 rpm for 2 min on glass slides. Cytospins were dried and stored at −80°C before use.

### Confocal microscopy

#### Patients and cytospin preparation

Peripheral blood (10 mL in EDTA) was obtained from a separate group of 20 metastatic breast cancer patients on progression before the initiation of a new line of treatment. Blood was collected by vein puncture after disposal of the first 5 mL in order to avoid contamination with epithelial cells from the patient skin during sample collection. Peripheral blood mononuclear cells (PBMC) were isolated after Ficoll-Hypaque (Sigma Life Science 10771) density gradient (d = 1.077 g/ml) centrifugation at 1800 rpm for 30 min, washed three times with PBS and centrifuged at 1500 rpm for 10 min. Aliquots of 500000 cells were centrifuged at 2000 rpm for 2 min on glass slides. Cytospins were dried and stored at −80°C for further use. All patients gave their written informed consent for their participation in this study, which has been approved by the Ethics and Scientific Committees of the University Hospital of Heraklion, Crete, Greece.

#### Immunofluorescence staining

A combination of direct and indirect immunofluorescence was used as previously described [11]. Cytospins were fixed with 4% formaldehyde in phosphate buffered saline (PBS) for 5 minutes at room temperature and permeabilized with Triton X-100. Fixed cells were incubated in blocking buffer (PBS, pH 7.4, 0.5% Triton X-100 and 1% fish skin gelatin) and stained indirectly with primary and then with secondary antibodies and directly with labelled primary antibodies. Primary antibodies for vimentin (Santa Cruz Biotechnology, sc-7558), CD45 [DakoCytomation, M 0701 (mouse) or Santa Cruz Biotechnology, sc-25590 (rabbit)], E-cadherin (BD Transduction Laboratories, 612130), fibronectin (BD Transduction Laboratories, 610077) and EpCAM (Acris Antibody AM10033 PU-N) and the corresponding anti-mouse and anti-rabbit secondary antibodies labeled with Alexa 488 (green staining, Invitrogen), Alexa 633 (blue staining, Invitrogen) and CF555 (red staining, Biotium) dyes were used.

In all experiments, we utilized anti-keratin 8/18/19 mouse monoclonal antibodies, A45-B/B3 (R002A, Micromet AG, Munich, Germany), used for CTCs analysis using CellSearch, A45-B/B3 antibodies were conjugated to Zenon 488 (green staining, Z25002, Molecular Probes), diluted 1/30 in blocking buffer without Triton X-100. Labelling of A45-B/B3 with Zenon 488 was performed following the instructions of the supplier.

The titration for optimal activities and the specificity of each antibody was evaluated using the different cell lines spiked in PBMCs from healthy patients. Specifically, we used the MCF-7 and T47D cell lines for the evaluation of anti-keratin, anti- E-cadherin and anti-EpCAM antibodies and the MDA.MB231 and Hs578T cell lines for the anti-vimentin and anti-fibronectin antibodies. In each separate immunofluorescence experiment, positive samples for epithelial and mesenchymal markers and negative controls prepared by omitting the respective primary antibody, to exclude non-specific binding, were included.

#### Identification of CTCs

All cytospin preparations of PBMCs were first examined under a conventional epifluorescence microscope (Leica) using 40 x objective lens with oil immersion and were further analyzed by confocal (Leica SP) microscopy. Keratins were labeled with anti-keratin 8/18/19 mouse monoclonal antibodies conjugated to Zenon 488 (green staining), vimentin was identified with anti-vimentin rabbit polyclonal antibodies and subsequently with secondary antibodies conjugated with CF 555 (red staining) and finally CD45 was labeled with mouse monoclonal antibodies followed by incubation with secondary antibodies conjugated with Alexa 633 (blue staining). To prevent any signal interference (green, red and blue) generated by the different emission spectra, the detection of each one of the markers was performed by sequential laser confocal scan. Fixed confocal settings were used for all specific measurements. Images were taken from all CTCs detected (DAPI positive and CD45 negative cells) and were stored electronically. As positive controls, cytospins of MCF-7 cells (keratin positive) or Hs578T cells (vimentin positive) spiked into normal donor PBMCs (CD45 and vimentin positive) were included in each separate experiment. Analysis of CD45 and keratin expression in CTCs and PBMCs revealed a highly significant difference between the 2 populations (Additional file 1).

### CellSearch analysis

#### Patients

Sixty-one patients with metastatic breast cancer with ≥2 CTCs per 7.5 ml of blood detected by the use of CellSearch were included in the current analysis. Patients were treated from 9/2007 to 10/2012 for metastatic breast cancer within prospective clinical trials organized by HORG (Hellenic Oncology Research Group) and had been assessed for the presence of CTCs before the initiation of first-line chemotherapy. The CellSearch Circulating Tumor Cell Kit (Veridex Warren, NJ) was used for CTC detection as previously described [12,13]. Patient data were prospectively obtained and retrospectively analyzed. All patients gave their written informed consent for their participation in the study, which has been approved by the Ethics and Scientific Committees of the University Hospital of Heraklion, Crete, Greece.

#### Breast cancer cell lines

For the calibration of keratin expression on CTCs, MCF-7 cells were spiked into 7.5 ml of peripheral blood obtained from healthy donors and were processed by the CellSearch System using the same protocol employed for patient samples [12,13].

### Image analysis

To quantify the fluorescence intensity of the markers of interest, images obtained from CellSearch or confocal microscopy were subjected to java-based image processing with the use of ImageJ program (NIH). CellSearch images from all CTCs detected in patient samples and representative images of 100 cells from MCF-7 cells were analyzed. Accordingly, images of all CTCs identified on patient cytospins and representative images of 100 cells from each MCF-7, T47D, MDA.231 and Hs578T cell lines obtained by confocal microscopy were also assessed by ImageJ. Fluorescence intensity was expressed as Corrected Total Cell Fluorescence (CTCF).

### Statistical analyses

*T*-test was used to compare 2 continuous variables. Pearson correlation and linear regression were used to assess correlation between continuous variables. One way ANOVA nonparametric test (Kruskal-Wallis) with Dunn’s post test was used to compare cell lines and CTCs. Overall survival (OS) was calculated from treatment initiation to death from tumor progression or death from any cause.

To examine the potential association of keratin expression on CTCs with patient outcome, the median keratin intensity on CTCs was determined using the values obtained from all CTCs detected by the use of the CellSearch System. Each individual CTC was classified as “high” or “low” according to the median keratin value; the keratin levels on CTCs were correlated with tumor characteristics. Patients with more than 50% of CTCs being “high” were characterized as “high keratin, HK”, whereas those with more than 50% of CTCs below the median value were designated as “low keratin, LK”. The two groups (HK and LK) were compared in terms of patient characteristics and overall survival.

All analyses were performed using the SPSS20 program.

## Results

### Immunofluorescence assay: intensity calibration and linearity of the detection system

**Figure 1:**
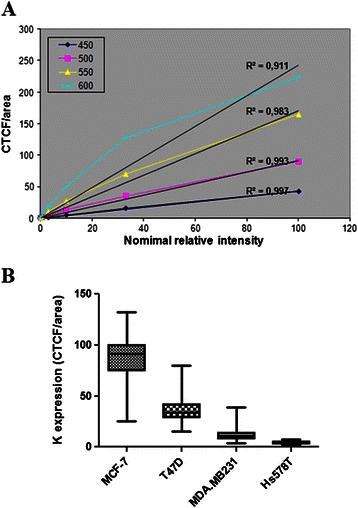
Linearity of the measured fluorescence intensity. **(A)** Corrected total fluorescence from fluorescent beads of different (100%, 33%, 10% and 3%) nominal fluorescence intensity was measured in different PMT settings (450, 500, 550 and 600 volts) and analyzed using ImageJ. Linear regression and R-square are shown for each PMT setting. **(B)** Box plot presenting the mean values (minimum to maximum) of keratin expression in different breast cancer cell lines. Fluorescence was measured in 550 volts and a total of 100 cells were evaluated for each cell line.

In order to establish an assay which would allow the effective measurement of fluorescence of epithelial and mesenchymal markers, we first determined the confocal settings in the range of which linearity of fluorescent measurements is maintained. For this purpose, we utilized beads of different fluorescence intensities (Focal Check Fluorescence Microscope Test Slide 1, F36909, Invitrogen), and captured a series of images at different laser settings. Fluorescence intensity was calculated with the use of ImageJ. Figure 1A shows the relative intensity curves obtained in different Photomultiplier (PMT) settings. Fluorescence intensity was practically linear up to 550 volts, indicating an effective and proportional measurement efficiency of both low and high intensity pixels under these adjustments. In order to assess whether the fluorescence intensity of pixels in cells under examination is included into the fluorescent limits of our standard curves, we analyzed the keratin expression in “epithelial” and “mesenchymal” breast cancer cell lines by measuring the mean intensity of the fluorescently labeled area of the cells (CTCF/area). Using cytospin preparations of MCF-7, T47D, MDA.MB231 and Hs578T cells, we found that the fluorescence values of all cells examined are distributed within the limits of the standard curve demonstrating that under these conditions low, moderate and high expression levels of keratin can be evaluated and compared (Figure 1B).

### Expression levels of epithelial and mesenchymal markers in breast cancer cell lines

**Figure 2:**
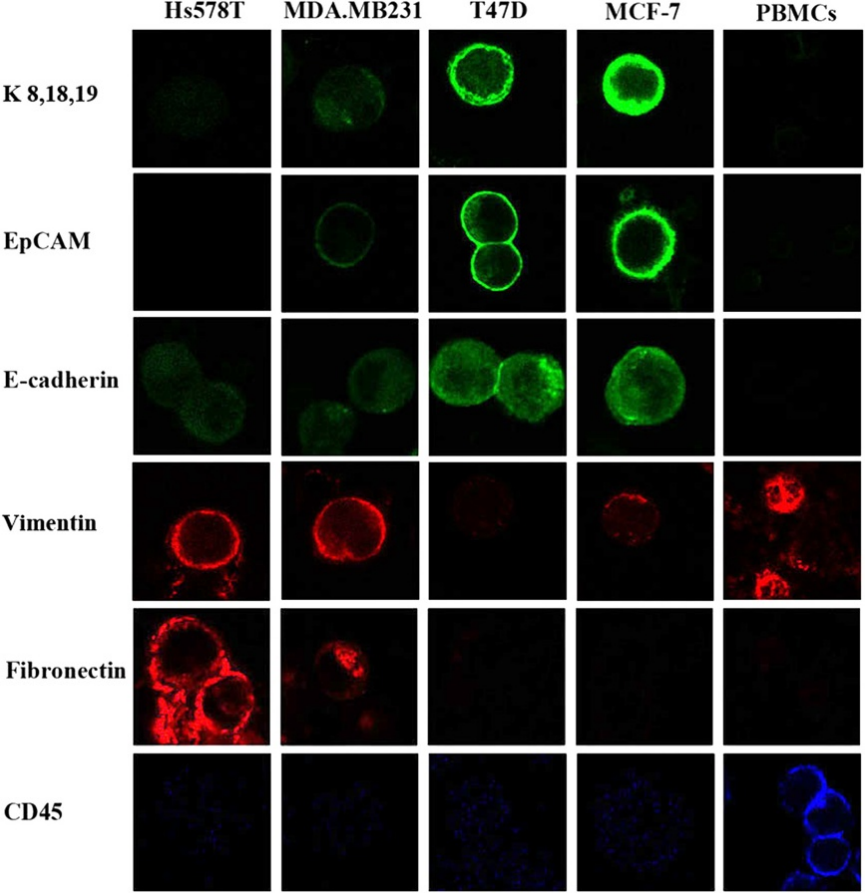
Expression patterns of epithelial and mesenchymal markers in breast cancer cell lines and PBMCs. Characteristic images of “epithelial” (MCF-7, T47D), “mesenchymal” (MDA.MB231, Hs578T) cells and PBMCs stained for epithelial (K, EpCAM and E-cadherin) markers (green) mesenchymal (vimentin and fibronectin) markers (red) and the leukocyte marker CD45 (blue).

**Figure 3:**
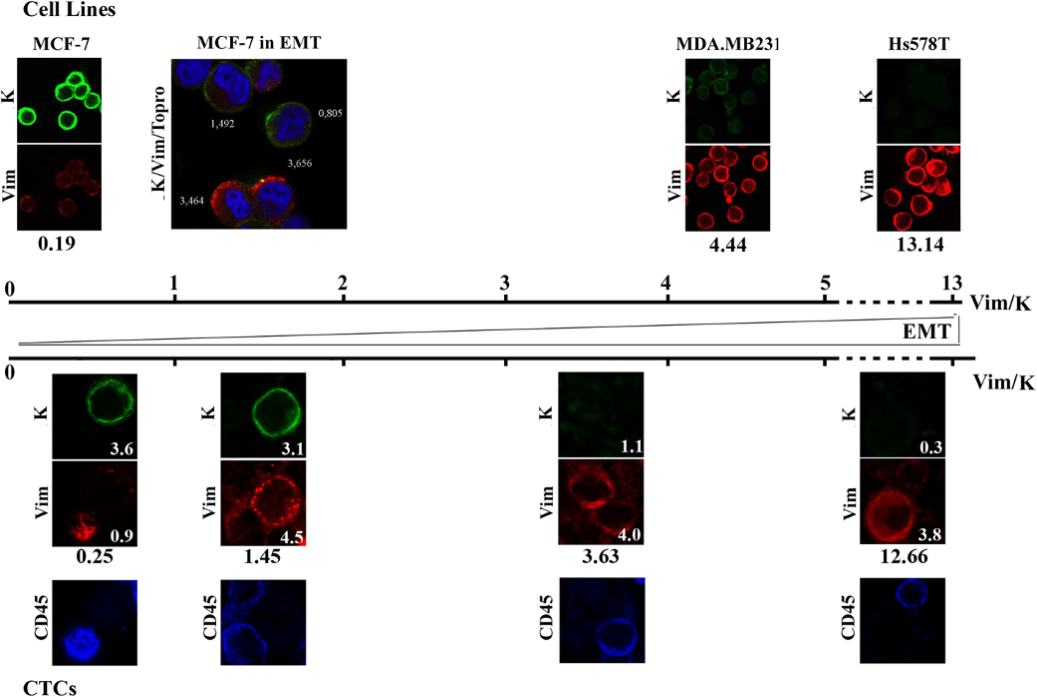
EMT status of breast cancer cell lines and CTCs. Cytospin preparations of cells stained for vimentin and K are placed along an axis with increasing vimentin/K (vim/K) ratios and EMT status. In the upper part of the figure are shown representative images of MCF-7, MDA.MB231 and Hs578T cells double stained for vimentin and K and a merge image of EGF treated MCF-7 cells stained for K (green), vimentin (red) and Topro (blue). Numbers shown below the images indicate the mean values of vim/K ratios calculated for each cell line. Numbers next to individual MCF-7 cells undergoing EMT are vim/K values measured for the indicated cells. Characteristic images from CTCs with different vim/K ratios and CD45 staining are presented in the lower part of the figure. Note the absence of CD45 staining in all CTCs and the presence of CD45 positive PBMCs (asterisks). Numbers shown inside the images, indicate the relative fluorescence intensity measured for each demonstrated marker, whereas numbers outside the images represent the vim/K ratios of the CTCs show.

The specificity of the antibodies used in our study and the pattern of epithelial and mesenchymal markers in breast cancer cell lines are presented in Figure 2, while the range and mean values calculated for epithelial (keratins, EpCAM) and mesenchymal (vimentin, fibronectin) markers in these cell lines are shown in Table 1 and Additional file 2. The calculated mean values demonstrate an upregulation of vimentin and fibronectin expression and downregulation of keratins and EpCAM in invasive cell lines (MDA.MB231, Hs578T), while poorly invasive cell lines (MCF-7, T47D) display the opposite profile. When the expression values were presented as a vimentin to keratin ratio, which we introduce as an EMT index, it was shown that the “epithelial” MCF-7 and T47D cell lines are characterized by low vim/K ratios (0.19 ± 0.05 for MCF-7 and 0.20 ± 0.07 for T47D cells), while “mesenchymal” MDA.MB231 and Hs578T cells display high vim/K (4.44 ± 1.98 and 13.14 ± 5.08, respectively) ratios (Table 1 and Figure 3). To further support the suggested correlation of a high vim/K ratio with a mesenchymal-like cell state, we examined the respective ratios in MCF-7 cells undergoing EMT. When MCF-7 cells were treated with EGF, most cells were characterized by variable vim/K ratios ranging from 0.45 to 5.05 with a mean value of 1.57 ± 1.02 (Table 1). Representative images are presented in Figure 3. In addition, we calculated the vimentin/EpCAM and fibronectin/K ratios in all cell lines examined. As shown in Additional file 2, the respective ratios displayed differences according to the ‘epithelial’ and ‘mesenchymal’ status of the cell lines with epithelial cell lines expressing lower ratios compared to the mesenchymal ones. Since K is broadly used for the identification of CTCs (by the use of immunofluorescence or the CellSearch System) whereas the wide range of the vim/K scale promoted a finer categorization of EMT in breast cancer cells, the generation of the EMT scale for the categorization of CTCs was based on the vim/K ratio.

**Table 1:** **Expression levels of epithelial and mesenchymal markers in breast cancer cell lines and CTCs**

		Keratin	vimentin	vim/K
**MCF-7**	Range	25.08 -131.69	9.01 – 24.75	0.12 - 0.49
	Mean	87.34 ± 18.99	15.68 ± 2.58	0.19 ± 0.05
**T47D**	Range	15.49 -80.36	2.80 -16.27	0.17 – 0.43
	Mean	35.43 ± 10.62	6.73 ± 2.16	0.19 ± 0.07
**MDA.MB231**	Range	4.22 -39.12	13.77 – 72.70	1.19 – 10.88
	Mean	11.47 ± 4.87	43.38 ± 12.06	4.44 ± 1.98
**Hs578T**	Range	1.73-7.40	12.30 -153.80	5.47 – 38.88
	Mean	4.02 ± 1.00	53.64 ± 24.81	13.14 ± 5.08
**MCF-7 EGF**	Range	7.59 – 43.06	11.79 – 52.23	0.45 – 5.05
	Mean	18.37 ± 7.56	23.63 ± 9.10	1.57 ± 1.02
**CTCs**	Range	1.49 – 213.19	0.00 - 155.24	0.00 – 22.46
	Mean	30.06 ± 25.00	26.42 ± 25.45	1.62 ± 3.96

The range and mean values (± SD) were calculated by measuring the fluorescence intensity (CTCF/area) of each marker. For the calculation of vim/K ratios, data were obtained from double staining (Keratin and vimentin) immunofluorescence experiment.

### Expression levels of epithelial and mesenchymal markers in CTCs

Twenty metastatic breast cancer patients evaluated before the initiation of a new line of treatment were screened for the presence of CTCs. A total of 110 CTCs detected in 5 patients with more than 2 CTCs per 10^6^ PBMCs were analyzed to determine the relative expression levels of keratin and vimentin. CTCs presented a significant heterogeneity in ratio values, ranging from 0.0 (cells without vimentin expression) to 22.46 (cells with almost exclusive vimentin expression). The mean value was 1.62 ± 3.96, compared to 0.12 ± 0.49 calculated for the “epithelial” MCF-7 cell line and 13.14 ± 5.08 for the “mesenchymal” Hs578Tcell line (Table 1 and Figure 3).

To define the epithelial or mesenchymal status of CTCs, the range of vim/K values calculated for MCF-7 and Hs578T cells, respectively, were used as cut-offs. Specifically, CTCs exhibiting ratios up to 0.49, representing the highest value of the ratio for MCF-7 cells, were characterized as “epithelial”, whereas values from 5.47 to 38.88, that correspond to the range calculated for Hs578T cells, defined “mesenchymal” CTCs. CTCs with values ranging from 0.49 – 5.46 were characterized as “intermediate” EMT undergoing cells. According to these cut-offs, 46% of CTCs could be classified as “epithelial” (with vim/K ratios ranging from 0.00-0.48), 5.4% (vim/K ratios ranging from 12.82-22.46) as “mesenchymal” and 48.2% of CTCs showing ratios between 0.57 and 3.35 as “intermediate” EMT undergoing CTCs. Moreover, 30% of all cells evaluated, exhibited lower keratin levels compared to “epithelial” luminal type breast cancer cell lines.

Furthermore, a significant inter- and intra-patient heterogeneity was evident regarding the EMT status of CTCs. The number of CTCs/10^6^ PBMCs detected in each patient as well as their distribution in “epithelial”, “intermediate” and “mesenchymal” phenotypes are included in [Additional file 3].

### Keratin levels of CTCs analyzed using the CellSearch platform and their association with tumor characteristics and clinical outcome of metastatic breast cancer patients

**Figure 4:**
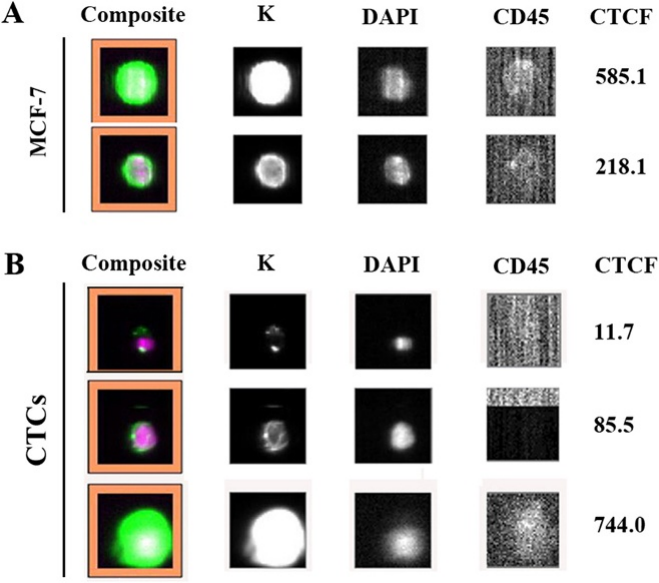
Expression levels of keratins in MCF-7 cells and CTCs analyzed using the CellSearch platform. Representative images and the corresponding CTCF values of MCF-7 cells spiked into blood from healthy donors **(A)** and CTCs **(B)**.

We sought to examine the significance of protein expression levels in CTCs detected by an approved method such as the CellSearch platform. To establish the methodology, we initially spiked MCF-7 cells into blood obtained from healthy blood donors and assessed keratin levels on images obtained using the CellSearch platform (Figure 4A) and by immunofluorescence analysis of cell cytospins. A strong correlation (R^2^ = 0.97) in the expression levels of keratin assessed by the use of the two approaches was evident (Additional file 4). Subsequently, we retrospectively measured keratin levels in 1262 CTCs identified in 61 patients with metastatic disease who had been evaluated before the initiation of first-line chemotherapy. Patient characteristics are listed in Table 2. Thirty-four (55.7%) patients were classified into the HK and 27 (44.3%) into the LK group according to the keratin expression levels on CTCs. A correlation was found between keratin levels and primary tumor characteristics. Low keratin levels were associated with triple negative status. Specifically, the mean keratin expression levels on CTCs detected in triple negative patients was 122.4 ± 99.98 compared to 175.0 ± 128.0 in the remaining patients (p < 0.0001, equal variance between the two groups, p = 0.056). Moreover, 72.7% of triple negative patients and 65% of ER-negative patients were classified as LK (Pearson correlation, p = 0.039 and p = 0.025, respectively). No difference in objective response to chemotherapy was evident according to keratin expression levels. A correlation was found for OS; 1-year OS was 73.3% and 46.2%, for patients in the HK and LK groups, respectively (Pearson correlation, p = 0.038).

**Table 2:** **Patients characteristics**

No of patients	61
**No of CTCs**	1262
**Age**	
Median (range)	62 (23–82)
	N (%)
**Menopausal status**	
Pre	17 (27.9)
Post	41 (67.2)
UN	3 (4.9)
**ER**	
Positive	38 (62.3)
Negative	20 (32.8)
UN	3 (4.9)
**PR**	
Positive	30 (49.2)
Negative	28 (45.9)
UN	3 (4.9)
**HER 2**	
Positive	14 (22.9)
Negative	44 (72.1)
UN	3 (4.9)
**Triple negative**	11 (18.0)
**Histology grade**	
1	1 (1.6)
2	16 (26.2)
3	26 (42.6)
UN	18 (29.5)
**No of CTCs/patients**	
Median (range)	2- 162
2-4	21 (34.4)
≥5	40 (65.6)

## Discussion

In the current study, we present data suggesting that keratin expression levels of EpCAM positive CTCs have potential clinical relevance and we propose a quantitative assay for the evaluation of the EMT status of CTCs, based on a mesenchymal to epithelial ratio calculated from the expression levels of vimentin and keratin measured on a single cell basis.

Differential gene expression levels of distinct keratins have been demonstrated among basal and luminal type breast cancer cell lines [1]. Thus, keratins 8 and 19 were significantly under-expressed in basal-like B as compared to basal-like A and luminal cell lines whereas, keratin 18 had significantly lower gene expression levels in all basal-like compared to luminal cell lines [1]. To our knowledge, our report is the first presenting data on protein expression levels in breast cancer cell lines and individual CTCs. In accordance to the report by Joosse et al. [1], keratin expression was higher in the luminal breast cancer cell lines MCF-7 and T47D compared to the basal-like B, MDA.MB231 and Hs578T cells. Interestingly, a subpopulation of CTCs, corresponding to 30% of all cells evaluated, exhibited lower keratin levels compared to “epithelial” luminal type breast cancer cell lines.

Using MCF-7 cells stimulated with EGF to induce EMT, we showed that keratin expression decreases under treatment. Moreover, the EMT status has been previously correlated with decreased levels of epithelial markers [14]. Accordingly, low keratin expression on CTCs could characterize CTCs undergoing EMT which theoretically are empowered with increased metastatic potential.

To have an insight into whether keratin expression, a potential surrogate marker for the EMT process, evaluated by a standardized and broadly available method such as CellSearch, could be related to clinical characteristics and patient outcome we retrospectively assessed expression levels on CTCs identified in a cohort of patients with metastatic breast cancer undergoing first-line chemotherapy. We demonstrated that low protein levels of keratins 8, 18 and 19 in EpCAM positive CTCs were associated with shorter OS. Low expression of keratins was also associated with triple negative histology indicating that low levels could predict for a more aggressive course of breast cancer [12,15,16]. Similarly, high mRNA expression of keratin 16 in metastatic breast cancer was associated with a shorter relapse-free survival when compared with patients with keratin 16 low expressing tumors [1]. Interestingly, keratin 16 upregulation is also a common phenomenon in basal-like breast cancer cell lines [1]. Data from both the study of Joosse et al. [1] and ours, although generated through different approaches, suggest that keratin levels do matter since they are associated with patient characteristics and clinical outcome. They also suggest that a potential surrogate marker for the EMT status of CTCs has clinical implications in metastatic disease.

The exclusion of EpCAM negative CTCs from CellSearch analysis remains a default setback of the CellSearch isolation methodology and could be compensated either with the acquisition of the cells that remain in the system or with the use of an EpCAM-independent isolation methodology. The study of EpCAM negative CTCs would be of interest in order to obtain a broader representation of the EMT grade in CTCs and its correlation with patient outcome.

We subsequently evaluated the combined relative expression of keratin and the mesenchymal marker vimentin as a means to refine our method regarding the characterization of the EMT status of CTCs. The EMT program is a highly dynamic process that involves a series of transitions and a spectrum of multiple intermediate states between the two extremes, the epithelial and the mesenchymal ones [17]. With the exception of a dual-colorimetric RNA–in situ hybridization assay for epithelial and mesenchymal transcripts defining various categories of the EMT process [5], no protein marker based quantifiable assays have so far been proposed for the characterization and evaluation of the mesenchymal and transitional EMT phenotypes of CTCs. Here, we established a simple and effective quantitative analysis of protein markers, utilizing data obtained through routine immunofluorescence analysis on CTCs non-selected according to EpCAM expression, thus expanding and complementing a previously established CTC identification methodology. More importantly, using the expression levels for mesenchymal (vimentin and fibronectin) and epithelial (keratin and EpCAM) markers of single cells, we introduced a numerical index for the determination of the EMT extent of CTCs. Subsequently, we applied expression levels for the generation of an EMT ‘gradient’ ranging from ‘epithelial’ to ‘mesenchymal’ rather than classifying cells into discrete categories. Since K is broadly used for the identification of CTCs (by immunofluorescence analysis or CellSearch) it was chosen for the generation of the EMT scale for the categorization of CTCs on the vim/K rather than the vimentin/EpCAM or fibronectin/keratin ratios. With the use of this index, we characterized each cell individually and positioned it onto this scale of increasing EMT status (see Figure 3) with a higher vim/K ratio suggestive of a stronger EMT phenotype. Interestingly, in agreement with recent studies [3-5] more than half of all CTCs detected in metastatic breast cancer patients presented an EMT phenotype of variable degree. Cells presenting differential EMT ratios, could be accredited with variable invasive capabilities. This is supported by recently reported data showing that the presence of mesenchymal markers on CTCs of metastatic breast cancer patients is an indicator of worse disease prognosis compared to the expression of keratins alone [18]. Moreover, the detection of a small percentage of purely mesenchymal CTCs in our study, is in accordance with previous reports [4,5,19], although an under-estimation of K positive CTCs could not be excluded because of the inefficiency of A45-B/B3 antibodies to recognize all types of keratins expressed in CTCs [1]. These cells could probably represent a highly invasive population and their presence further supports the view that CTCs cannot be effectively evaluated when their isolation and detection is based on epithelial markers alone.

A limitation of our study is that due to the retrospective nature of the analysis on CellSearch data, the EMT index could not be generated and validated. On the other hand, due to the small number of patients evaluated for the EMT ratio on CTCs, we cannot comment on the clinical significance of this approach. However, it represents a simple, practical and cost-effective methodology, which can easily be exported due to the wide use of immunofluorescence analysis for the detection of CTCs and for which we consider that it merits further evaluation as a prognostic tool.

## Conclusions

Our study highlights the significance of quantifying protein expression for the characterization of CTCs. Data from CellSearch analysis revealed a correlation between the keratin levels of CTCs, the tumor characteristics and outcome of patients with metastatic breast cancer. By evaluating the relative vimentin and keratin expression levels of unselected, immunofluorescently labeled CTCs on cytospins, we generated a numerical index on which we based the establishment of an EMT hierarchy ‘gradient’ ranging from ‘epithelial’ to ‘mesenchymal’. This approach could offer significant prognostic information upon diagnosis or during follow up of patients with breast cancer. Although this method could be easily applied following detection of CTCs using immunofluorescence, we are currently developing an automated methodology for the detection, quantification and analysis of the expression levels of different protein markers to reflect their heterogeneous biological properties.

## Additional files

Additional file 1: **Keratin and CD45 expression levels in CTCs and PBMCs.** Description: Column bar graph presenting the mean values (±SD) of CD45 (panel A) and keratin (panel B) in CTCs and PBMCs. Expression levels were calculated in all CTCs found (110 cells) and an equal number of PBMCs by measuring the fluorescence intensity (CTCF/area) of each marker. T- test statistical analysis was performed among the two populations (for both panels p < 0001). CD45 expression ranged between 29.49 to 171.5 (±31.87) and between 0.0 to 13.30 (±3.97) for PBMCs and CTCs respectively. Keratin expression levels ranged from 0.11 to 3.86 (±0.96) and from 3.00 to 155.0 (±27.65) for PBMCs and CTCs respectively.
Additional file 2: **Mesenchymal/epithelial ratios in breast cancer cell lines.** Description: The range and mean values (± SD) were calculated by measuring the fluorescence intensity (CTCF/area) of each marker. For the calculation of vimentin/EpCAM and fibronectin/K ratios, data were obtained from double staining (EpCAM and vimentin or K and fibronectin respectively) immunofluorescence experiment.
Additional file 3: **Distribution of various CTC phenotypes in breast cancer patients.** Description: Number (No) of CTCs detected in 10^6^ PBMCs for each patient and their percent distribution in “epithelial”, “intermediate”, “mesenchymal” phenotypes according to their vim/K ratios.
Additional file 4: **Expression levels of keratin after confocal microscopy or CellSearch analysis.** Description: Scatterplot with a regression line showing a strong correlation between the keratin expression levels (CTCF) of MCF-7 cells (85 in total) measured after analysis with CellSearch or immunofluorescence confocal microscopy.

## Abbreviations

CTCs: Circulating tumor cells
EMT: Epithelial-to-Mesenchymal Transition
OS: Overall survival
K: Keratin
PMT: Photomultiplier
MET: Mesenchymal to Epithelial Transition
HK: High keratin expression of CTCs
LK: Low keratin expression of CTCs
vim/K: Vimentin/keratin
