# Supplementary figures and images for: Variable expression levels of keratin and vimentin reveal differential EMT status of circulating tumor cells and correlation with clinical characteristics and outcome of patients with metastatic breast cancer
Source: BMC Cancer. 2015 May 13;15:399. doi: 10.1186/s12885-015-1386-7 (PMC4434869; doi:10.1186/s12885-015-1386-7)

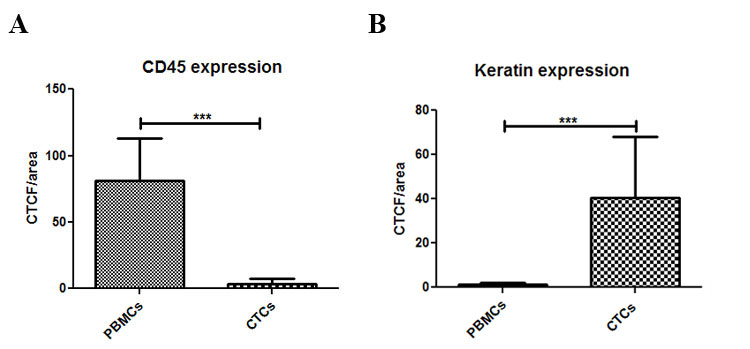

Supplement: Additional file 1: — Keratin and CD45 expression levels in CTCs and PBMCs. Description: Column bar graph presenting the mean values (±SD) of CD45 (panel A) and keratin (panel B) in CTCs and PBMCs. Expression levels were calculated in all CTCs found (110 cells) and an equal number of PBMCs by measuring the fluorescence intensity (CTCF/area) of each marker. T- test statistical analysis was performed among the two populations (for both panels p < 0001). CD45 expression ranged between 29.49 to 171.5 (±31.87) and between 0.0 to 13.30 (±3.97) for PBMCs and CTCs respectively. Keratin expression levels ranged from 0.11 to 3.86 (±0.96) and from 3.00 to 155.0 (±27.65) for PBMCs and CTCs respectively. [file 12885_2015_1386_MOESM1_ESM.jpeg]

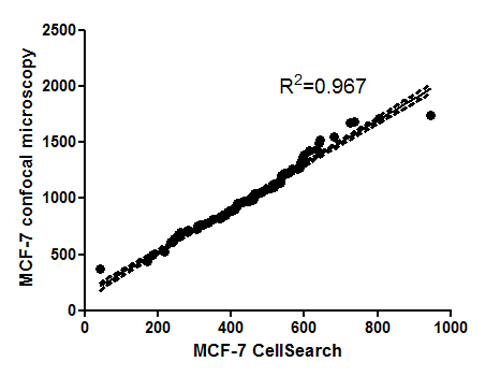

Supplement: Additional file 4: — Expression levels of keratin after confocal microscopy or CellSearch analysis. Description: Scatterplot with a regression line showing a strong correlation between the keratin expression levels (CTCF) of MCF-7 cells (85 in total) measured after analysis with CellSearch or immunofluorescence confocal microscopy. [file 12885_2015_1386_MOESM4_ESM.jpeg]
