# Supplementary material for: Variable expression levels of keratin and vimentin reveal differential EMT status of circulating tumor cells and correlation with clinical characteristics and outcome of patients with metastatic breast cancer
Source: BMC Cancer. 2015 May 13;15:399. doi: 10.1186/s12885-015-1386-7 (PMC4434869; doi:10.1186/s12885-015-1386-7)
Supplement: Additional file 2: — Mesenchymal/epithelial ratios in breast cancer cell lines. Description: The range and mean values (± SD) were calculated by measuring the fluorescence intensity (CTCF/area) of each marker. For the calculation of vimentin/EpCAM and fibronectin/K ratios, data were obtained from double staining (EpCAM and vimentin or K and fibronectin respectively) immunofluorescence experiment. [file 12885_2015_1386_MOESM2_ESM.pdf]

Additional file 2

Title: Mesenchymal/epithelial ratios in breast cancer cell lines

Description: The range and mean values (± SD) were calculated by measuring the fluorescence intensity (CTCF/area) of each marker. For the calculation of vimentin/EpCAM and Fibronectin/K ratios, data were obtained from double staining (EpCAM and vimentin or K and Fibronectin respectively) immunofluorescence experiment.

		Vimentin/EpCAM	Fibronectin/K
**MCF-7**	Range	0.13 -0.49	0.01–0.07
	Mean	0.26 ± 0.10	0.037 ± 0.016
**T47D**	Range	0.19-0.57	0.01-0.05
	Mean	0.35 ± 0.10	0.022 ± 0.009
**MBA.MB231**	Range	4.33-51.22	7.00-47.00
	Mean	18.55 ± 11.36	17.12 ± 11.60
**Hs578T**	Range	EpCAM not detectable	113.00-304.00
	Mean	EpCAM not detectable	206.78 ± 53.19
