# Supplementary material for: Variable expression levels of keratin and vimentin reveal differential EMT status of circulating tumor cells and correlation with clinical characteristics and outcome of patients with metastatic breast cancer
Source: BMC Cancer. 2015 May 13;15:399. doi: 10.1186/s12885-015-1386-7 (PMC4434869; doi:10.1186/s12885-015-1386-7)
Supplement: Additional file 3: — Distribution of various CTC phenotypes in breast cancer patients. Description: Number (No) of CTCs detected in 106 PBMCs for each patient and their percent distribution in “epithelial”, “intermediate”, “mesenchymal” phenotypes according to their vim/K ratios. [file 12885_2015_1386_MOESM3_ESM.pdf]

Additional file 3 [Additional File3.pdf]

Title: Distribution of various CTC phenotypes in breast cancer patients

Description: Number (No) of CTCs detected in 10^6 PBMCs for each patient and their percent distribution in “epithelial”, “intermediate”, “mesenchymal” phenotypes according to their vim/K ratios.

**Patients**	**No of CTCs**	**“Epithelial”**	**“Intermediate”**	**“Mesenchymal”**
P1	4	50	50	0
P2	5	80	20	0
P3	12	42	58	0
P4	55	29	60	11
P5	34	70	29	0
